# Validation of the translated Negative Physical Self Scale in a sample of Asian women living in Canada

**DOI:** 10.1371/journal.pone.0301184

**Published:** 2024-05-02

**Authors:** Shahrazad Amin, May Ly, Kaylee Misener, Natalie Brown, Maya Libben

**Affiliations:** 1 Department of Psychology, University of British Columbia, Vancouver, British Columbia, Canada; 2 Department of Psychology, University of Regina, Regina, Saskatchewan, Canada; 3 Department of Psychology, University of British Columbia, Okanagan, Kelowna, British Columbia, Canada; St John’s University, UNITED STATES

## Abstract

**Objectives:**

Body dissatisfaction is often linked to the internalization of Western beauty standards. Existing measures of body dissatisfaction, developed in Western societies, may fail to capture complex variations across ethnicities and cultures. The Negative Physical Self Scale (NPSS) assesses cognitive, affective, perceptual, and behavioural facets of body dissatisfaction. While unique in its consideration of Chinese ideals of body image, the NPSS has recently been translated and validated in a North American sample. The English-translated version of the NPSS has the potential to be an appropriate body dissatisfaction assessment tool for Asian women living in North America. The current study aims to validate the NPSS in an Asian female population living in Canada.

**Methods:**

A sample of 899 undergraduate women residing in Canada with self-identified Asian ethnicity completed an online survey consisting of the NPSS and other measures of body dissatisfaction.

**Results:**

An initial confirmatory factor analysis indicated that the four-factor structure of the NPSS, previously suggested in a primarily non-Asian North American sample, was a poor fit for the data. A second-order multidimensional model, based on a model proposed during the original development of the NPSS in a Chinese sample, indicated good fit once items were removed due to loadings < .60. High internal consistency between subscales and strong convergent validity with other measures were demonstrated. Notably, the NPSS Body Concern subscale demonstrated high convergence with other popular measures of body dissatisfaction and has the potential for use as a brief measure of body dissatisfaction among North American Asian females in clinical and research settings.

**Conclusions:**

The NPSS provides a valid assessment of body dissatisfaction among a sample of Asian women living in Canada, a specific subpopulation that has not been previously investigated. The findings highlight the importance of developing culturally sensitive measures of body dissatisfaction for differing ethnic and cultural groups.

## Introduction

Body dissatisfaction (BD) is a highly prevalent phenomenon characterized by individuals’ discontent with their body size, shape, and weight [1–7]. Beyond its immediate implications, BD assumes a pivotal role within the diagnostic criteria of diverse eating disorders, including anorexia and bulimia nervosa [8]. Additionally, BD predicts the maintenance of eating disorder pathology [9–11], a relationship that is notable across cultures [6, 12]. Given the clear association between BD and mental health outcomes on a global level, it is important to have appropriate cross-cultural measures of body image and BD [9, 13].

Numerous dimensions of BD have been identified including facets of perception, cognition, and behavior [14]. Cultural influences, such as parental remarks, cultural ideals, and media portrayal of thin women, can significantly influence BD [6, 15, 16]. For instance, Asian American women who internalize media-based beauty ideals tend to experience greater BD [17, 18]. Moreover, evidence suggests that individuals from low socioeconomic status countries tend to prefer larger bodies compared to those from high socioeconomic status countries [6]. Among immigrant women, body image is further influenced by acculturation, which can cause women to idealize beauty standards from both their native culture and the culture of their new country of residence [16].

Several scales have been developed to evaluate BD in research and clinical settings [19–25]. For example, the Body Shape Questionnaire (BSQ) [2], the Eating Disorder Examination Questionnaire (EDE-Q) [26], and the Eating Attitudes Test (EAT) [27] target underlying cognitive-affective and behavioral symptoms related to body weight and shape. However, as these scales were developed in Western, high-income, English-speaking countries, some of the content and language of these measures are specific to Western culture and fail to capture the complexity of contextual variations found across cultures [28]. Given the lack of culturally specific scales for body image concerns, there is a growing field of research supporting the development and validation of culturally and ethnically appropriate BD scales.

In an effort to better understand body image concerns specific to Asian individuals, Chen and colleagues [20] developed the Negative Physical Self Scale (NPSS). The NPSS is a 48-item multidimensional measure of BD for Chinese adolescents and adults. The subscales encompass concerns related to fatness, height, facial appearance, and general appearance. The NPSS is unique in that it considers cultural ideals related to body image, specifically factors present in Chinese collectivist culture. For example, concerns related to weight, such as identifying as being too slim, have been prevalent in China due to socio-cultural implications stemming from the historical relationship between poverty and low weight [29]. Another culturally influenced facet of BD, featured in the NPSS, is a desire to be taller for both men and women. It has been proposed that the focus on height in China may be due to the cultural perception that height corresponds with authority and positions of power [30]. Another aspect unique to Eastern measures of body image, like the NPSS, is a greater emphasis on facial features in judging one’s attractiveness, making facial appearance a significant factor of concern for BD. For instance, Chinese culture prioritizes facial symmetry as well as eyebrow, eye, nose, and mouth shape as important factors in determining beauty [29]. As such, the factors of Fatness, Thinness, Shortness, and Facial Appearance were identified by Chen and colleagues [20] as potential contributors to BD, and thereby were incorporated into the NPSS. Over the course of four studies, Chen et al. [20] examined the NPSS using over 3,000 male and female participants aged 12–24 years. Five underlying dimensions of the NPSS were identified, with General Appearance, Fatness, and Thinness aligning with existing Western studies of BD (i.e., the Physical Self-Perception Profile [31], the Eating Disorder Inventory [32] and the Body Esteem Scale for Adolescents and Adults [33]). The two factors that did not align with Western measures were Shortness and Facial Appearance, indicating that these were aspects of BD uniquely measured by the NPSS. While Western measures of BD are often the standard in research, Chen et al.’s results [20] highlight that Western measures may fail to capture constructs that are central to BD across diverse cultures. Given the discrepancies between measures of Western and Asian BD, there is an evident benefit in the use of the NPSS as a culturally specific body image scale.

The NPSS has been widely used in studies of BD among Chinese individuals, predicting attentional bias, self-esteem, and eating disorder symptomatology [34–42]. Furthermore, Ly and colleagues [43] (see also [44]) developed and assessed an English translation of the NPSS in a North American female sample that was primarily Caucasian (63%), with only 5% of participants identifying as Asian (the remaining sample was comprised of participants who identified as Indigenous (2%), Black (<1%), other (29%), or did not specify (<1%)). Convergent validity was robust, aligning the English NPSS with two Western scales measuring body image and eating concerns—the BSQ and EDE-Q. Interestingly, Ly et al. [43] found that the five-factor structure reported in the original Chinese NPSS sample [20] did not replicate within a primarily Caucasian North American female cohort. Specifically, the authors reported a four-factor model where three factors (Thinness, Shortness, and Facial Appearance) closely resembled the original NPSS subscales and the fourth factor amalgamated all items from the Fatness and General Appearance subscales, along with one item from the Facial Appearance subscale. This discrepancy was attributed to potential disparities in body image ideals between the two cultures and differences in the ethnic makeup of the samples. Ly et al.’s findings [43] are consistent with prior research demonstrating the non-replicability of factor structures in body image scales between Eastern and Western populations [28, 45, 46].

Recently, Wang and colleagues [47] investigated the factor structure and measurement invariance of the English NPSS among North American men across three broad ethnic groups–Caucasian (46%), Asian (19%) and Other (33%). Results supported a four-factor structure with three sub dimensions and partially replicated both Ly et al.’s [43] and Chen et al.’s [20] prior results. Interestingly, the authors found that the factor structure and factor loadings of the NPSS were equal across ethnic groups. In other words, the BD constructs measured by the NPSS demonstrated the same meaning across men from different ethnic groups residing in North America, suggesting potential acculturation effects in shaping one’s body ideals. The translation of these effects to a female population remains uncertain, given that levels of body dissatisfaction tend to be higher among females in comparison to males and are predominantly characterized by distinct features [15]. Notably, research on gender-specific body dissatisfaction has revealed that males often desire increased height and strength, whereas females commonly aspire to achieve a thinner physique [29].

Despite ample evidence underscoring the influence of culture and ethnicity on BD, there is currently no validated BD measure tailored specifically for Asian-identifying women residing in North America. This is particularly pertinent given that Western ethnic minority groups often navigate a unique convergence of diverse cultural and ethnic norms. Currently, it is unclear whether BD constructs for Asian women residing in North America would resemble those of Caucasian women (as suggested by Wang et al.’s [47] results among men) or would show differing constructs (similar to Chen et al. [20] vs. Ly et al.’s [43] contrasting results). Asian Americans and Asian Canadians represent substantial ethnic minorities in both countries; understanding and properly assessing BD among this population is critical for effective prevention and treatment of BD-related issues. Despite the apparent suitability of the English NPSS for this demographic, research investigating its application to Asian women in North America has yet to emerge. The current study aimed to examine the factor structure and measurement invariance of the English-translated version of the NPSS using a sample of women with self-identified Asian ethnicity currently residing in Canada. Building upon extant research, we investigated two plausible structural models. First, we assessed the viability of the four-factor model (Body Concern, Thinness, Shortness, and Facial Appearance) proposed by Ly et al. [43]. Second, we evaluated a multidimensional second-order structural model predicated on a four-factor framework with three sub-dimensions (cognition-affect, behavior, and projection) identified by Chen et al. [20]. Finally, we examined the internal consistency, convergent validity, and incremental validity of the test scores of the NPSS test scores, aiming to enhance the understanding of the scale’s psychometric properties in this specific population.

## Method

### Participants

The sample was comprised of 899 women between the ages of 18 and 25 (*M*_*age*_ = 19.60, *SD* = 1.49). All participants self-identified as either East Asian, South Asian, or Southeast Asian and lived in Canada at the time of participation. Participants were recruited from the University of British Columbia (Vancouver and Okanagan campuses) and were enrolled in a psychology course that offered course credit for research participation. Written consent was obtained from all participants. Study procedures were approved by the Behavioural Research Ethics Board at the University of British Columbia Okanagan (H17-02395). Data collection occurred between 2019 and 2021. Place of origin and number of years lived in North America are included in Table 1.

**Table 1:** Participant place of origin and time lived in North America.

	*n*	%
Place of origin		
Canada	262	29
United States	19	2
Europe	1	<1
Middle East	12	1
Asia	599	67
Africa	2	<1
South America	2	<1
Australia	2	<1
Number of years lived in North America		
Born in North America	281	31
0–5 years	349	39
6–10 years	64	7
11–15 years	48	5
16 or more years	50	6
Not specified	107	12

### Measures

#### NPSS

The Negative Physical Self Scale (NPSS) [20] is a 48-item self-report measure designed to measure cognitive, affective, perceptual, and behavioral components of BD. Items are assessed on a 5-point Likert scale ranging from 0 (*never*) to 4 (*always*) and constitute five subscales: General Appearance, Facial Appearance, Fatness, Thinness, and Shortness, which all measure self-perceptions of physical appearance. The current study used the validated English version of the NPSS [43]. Subscale scores are the average of the summed responses. Total scores represent the sum of the subscale scores. A higher score is indicative of a more negative perception of body image.

#### BSQ

The Body Shape Questionnaire (BSQ-34) [2] investigates cognitions, perceptions, and concerns surrounding body image. In this 34-item scale, participants respond to questions regarding body image cognitions and behaviors on a 6-point numerical scale with greater scores indicating higher concerns regarding body shape and weight. The BSQ has been validated and found to have good test-retest reliability (three weeks) with a coefficient of *r* = 0.88, *p* < .001 [48]. While explicit validation studies investigating the psychometric properties of the BSQ among Asian North American females are lacking, this measure has been used frequently among Asian American females [49–52] and a translated version has been validated among young adult Korean women [53].

#### EDE-Q

The Eating Disorders Examination—Questionnaire 6.0 (EDE-Q) [26] is a self-report scale consisting of 28 items that measure the extent of certain cognitive symptoms and behavioral issues found in eating disorders. Items are assessed on a 7-point numerical scale ranging from 0 (*no days*) to 6 (e*very day*) and constitute four subscales: Restraint, Eating Concern, Shape Concern, and Weight Concern. The global score is the average of the sum of the four subscales, with higher scores indicating a higher level of eating disorder symptomatology. The frequency of compensatory eating behaviors is also measured by having respondents indicate how many days, of the past 28 days, they engaged in a specific behavior, such as how many times a participant lost control when eating or used laxatives to control their shape or weight. The EDE-Q demonstrates good test-retest reliability with *r* coefficients ranging from 0.81 to 0.94 where *p* < .001 [54]. The EDE-Q is used in both clinical settings and research settings [55]. Although a validity study investigating the psychometric properties of the EDE-Q among Asian North American females is lacking, recent studies suggest that it is a sensitive measure to eating pathology among female Asian Americans [56, 57]. Furthermore, in a non-North American Asian sample, Nakai et al. [58] evaluated the psychometric properties of the EDE-Q on Japanese women and reported good internal consistency for the scores of all four EDE-Q subscales: Restraint (*α*  =  0.74), Eating Concern (*α*  =  0.75), Shape Concern (*α*  =  0.89) and Weight Concern (*α*  =  0.80).

#### Procedure

All data were collected using the Qualtrics online survey platform (Qualtrics, Provo, UT). Participants completed an online consent form prior to proceeding to the online questionnaire. Participants were asked for demographic information and subsequently completed a battery of questionnaires that included the NPSS, BSQ, and EDE-Q.

### Data preparation

As part of a larger study examining the utility of the NPSS in various populations, an initial sample of 2,859 participants enrolled in the study. For the current study, participants were removed if they did not meet the study eligibility criteria (i.e., lived in North America, identified as female, identified as Asian and aged 18–25), had missing data (i.e., did not complete the survey), and/or failed the attention check question. The final sample consisted of 899 female undergraduates.

### Statistical analyses

A confirmatory factor analysis [59] was initially performed on the 48 items of the NPSS based on the four-factor structure described by Ly and colleagues [43]. Subsequently, a second-order multidimensional model [59] based on the original NPSS scale development study [20] was tested. In this model, the four factors (i.e., Body Concern, Thinness, Shortness, Facial Appearance) were second-order factors, and each consisted of three subdimensions (i.e., cognition-affect, behavior, projection) as first-order factors [59]. Internal consistency was examined using Cronbach’s alpha (α) and McDonald’s omega (ω). Although Cronbach’s alpha is the most widespread measure of internal consistency, several of its assumptions are often violated. Recent literature has recommended the use of McDonald’s ω as an alternative to α [60]. Correlational analyses were used to determine intercorrelations between subscales and convergent validity with the BSQ, EDE-Q, and NPSS [61]. Correlations between body mass index (BMI) and other scales were also measured. BMI was calculated according to the World Health Organization criteria using the following formula: BMI = (weight in kg)/(height in meters)^2^. Participants’ height and weight values were self-reported. Hierarchical regression analysis was used to examine incremental validity of the NPSS [61].

## Results

### Confirmatory factor analysis

In accordance with Wang et al. [47], the criteria for good model fit was based on the following: χ^2^/df ≤ 3.0 [62], Root-mean-square error of approximation (RMSEA) ≤ .80 [63], Tucker–Lewis index (TLI) ≥ .90 [62], Comparative fit index (CFI) ≥ .90 [62], and Standardized root-mean-square residual (SRMR) ≤ .08 [64].

In Model 1, an initial CFA was conducted on the four-factor model found by Ly et al. [43]. Each factor represented a domain of BD, namely Body Concern, Thinness Concern, Shortness Concern, and Facial Appearance Concern. A statistically significant chi-square test, χ^2^(1074) = 8746.13, *p* < .001, indicated poor model fit, however, this index is sensitive to sample size. Looking at other fit indices (e.g., CFI) aids in interpreting model fit. Results indicated that not all fit indices supported this four-factor model, where χ^2^/*df* = 8.14, CFI = .75, TLI = .74, RMSEA = .09.

A second confirmatory factor analysis (Model 1.1) was performed on the NPSS after items with loadings < .60 were removed sequentially from the model [59]. In total, seven items were removed (items 4, 5, 9, 10, 25, 38, 45). Item 4 belonged to the Shortness subscale, items 5 and 10 belonged to the Thinness subscale, items 9 and 38 belonged to the Facial Appearance subscale, item 25 belonged to the Fatness subscale, and item 25 belonged to the Body Concern subscale. A statistically significant chi-square test, where χ^2^(773) = 6445.47, *p* < .001, indicated poor model fit. Most fit indices slightly improved but still suggested poor fit to the data, where χ^2^/*df* = 8.34, CFI = .79, TLI = .78, RMSEA = .09.

Due to the poor fit of the 4-factor model, a second-order multidimensional model was tested based on Chen et al. [20]. In this model (Model 2), the four factors (i.e., Body Concern, Thinness, Shortness, Facial Appearance) were second-order factors, and each consisted of three subdimensions (i.e., cognition-affect, behavior, projection) as first-order factors. A CFA was conducted on the multidimensional model described above. Fit indices improved noticeably, where χ^2^(1062) = 4810.04, *p* < .001, χ^2^/*df* = 4.53, CFI = .88, TLI = .87, RMSEA = .06. However, some indices (i.e., CFI, TLI) still did not meet criteria for good fit (Table 2).

**Table 2:** NPSS confirmatory factor analysis fit and error indexes.

Model	*χ* ^*2*^	*df*	*χ* ^*2*^ */df*	*p*	RMSEA	CFI	TLI	SRMR
1	8746.13	1074	8.14	.00	.09	.75	.74	.08
1.1	6445.47	773	8.34	.00	.09	.79	.78	.07
2	4810.04	1062	4.53	.00	.06	.88	.87	.07
2.2	3897.65	968	4.03	.00	.06	.90	.90	..07

*Note*. Model 1 = four-factor model; Model 1.1 = modified four-factor model; Model 2 = second-order model; Model 2.2 = modified second-order model

As such, another CFA was conducted on the multidimensional model (Model 2.1), where items loading below < .60 were removed sequentially from the model. In total, 2 items were removed (Items 10 and 19). This is consistent with Wang et al. [47], in which Item 19 was removed from the model due to a loading below < .60. This model had good fit, where χ^2^(968) = 3897.65, χ^2^/*df* = 4.03, CFI = .90, TLI = .90, RMSEA = .06 (Table 2).

### Internal consistency

An analysis of the reliability of the four subscales of the NPSS was conducted to assess internal consistency. Similar to the findings of Ly and colleagues [43], the subscales demonstrated excellent internal consistency. The internal consistencies of the subscales were measured with Cronbach’s alpha (α), where the Body Concern, Thinness, Shortness, and Facial Appearance subscales were 0.93, 0.90, 0.91, and 0.86, respectively. McDonald’s omega (ω) measured 0.92 (Body Concern), 0.90 (Thinness Concern), 0.91 (Shortness), and 0.85 (Facial Appearance). Each of the subscales demonstrated higher internal consistency in comparison to the original scale validation by Chen and colleagues [20]. The internal consistency of the BSQ and EDE-Q was also examined, with values of α = 0.97 and α = 0.95 respectively.

### Intercorrelations

Intercorrelations between factors of the NPSS are presented in Table 3. All subscales were significantly correlated with each other. Consistent with Ly and colleagues [43], the Facial Appearance subscale had the strongest relationship with the Body Concern subscale (*p* < .001), suggesting that both subscales measure a similar construct related to negative self-image. Additionally, the Thinness subscale demonstrated a negative relationship with the Body Concern subscale, suggesting that increased thinness may be associated with less body dissatisfaction. The Shortness subscale had the weakest correlation with the Body Concern subscale, suggesting that height may not be as relevant to body dissatisfaction. Intercorrelations are presented in Table 3.

**Table 3:** Intercorrelations between the factors of the NPSS.

	Body	Thinness	Shortness	Facial Appearance
Body				
Thinness	-.30**			
Shortness	.12**	.21**		
Facial Appearance	.51**	.16**	.23**	

*Note*. ** *p* < .01

### Convergent validity

The convergent validity of the NPSS was measured alongside other well-established measures of BD and disordered eating in the literature. Using Pearson’s *r*, two-tailed correlations between the NPSS, BSQ, EDE-Q, and the participants’ BMI were examined (Table 4). In accordance with Ly and colleagues [43], the scores of the total NPSS had large positive correlations with the total scores of the BSQ and EDE-Q in the full sample. However, it should be noted that the correlations presented in Table 4 were weaker than those reported by Ly and associates [43]. These findings suggest that the NPSS measures theoretically similar constructs to the BSQ and EDE-Q, in this case, BD and eating disorder pathology, respectively. Finally, when interpreting the values in Table 4, it is important to consider that higher scores on the NPSS subscales reflect *dissatisfaction* with physical characteristics (i.e., dissatisfaction with thinness (e.g., “The people that I like think I am too thin”), dissatisfaction with shortness (e.g., “I think I am too short”) and dissatisfaction with facial appearance (e.g., “People around me do not like the way my face looks”).

**Table 4:** Correlations between the extracted NPSS subscales, BSQ, EDE-Q, BMI, and years lived in North America.

	Body Concern	Thinness	Shortness	Facial Appearance	NPSS Total
BSQ Total	.74**	-.09**	.21**	.51**	.43**
EDE-Q Restraint	.64**	.04	-.01	.02	.02
EDE-Q Eating Concern	.63**	.01	.04	.14**	.16**
EDE-Q Shape Concern	.81**	-.01	.07	.13**	.13**
EDE-Q Weight Concern	.78**	-.19**	.13**	.48**	.49**
EDE-Q Total	.79**	.05	.01	.04	.05
BMI	.38**	-.39**	-.09**	.07*	.28**
Years in North America	.05	.04	.02	.05	.08

*Note*. * *p* < .05.

** *p* < .01

### Incremental validity

Incremental validity of the NPSS was assessed using hierarchical regression analysis to predict EDE-Q scores. The assumptions of homoscedasticity, no multicollinearity, and independent errors were all met prior to performing the hierarchical regression analysis. With BMI and BSQ total scores entered as independent variables, the first model accounted for 69.9% of the variance in EDE-Q scores, *F*(2, 839) = 973.44, *p* < .001. When NPSS subscales were entered, the second model accounted for 74.8% of the variance in EDE-Q scores, *F*(4, 835) = 40.46, p < .001. The NPSS accounted for an additional 4.9% of the variance in EDE-Q scores, which is small but significant. Of the NPSS subscales, Body Concern was the only subscale that significantly contributed to the model. This is consistent with Ly and colleagues’ [43] findings where Body Concern demonstrated the strongest contribution to the model. Shortness, Thinness, and Facial Appearance may not be as relevant to EDE-Q scores as Body Concern. Results from the regression analysis are displayed in Table 5.

**Table 5:** Hierarchical regression analysis in predicting EDE-Q from BMI, BSQ, and NPSS.

	*B*	*SE*	β	R^2^	ΔR^2^
Step 1				.70	
BMI	.04	.00	.19***		
BSQ	.93	.02	.80***		
Step 2				.75	.05
BMI	.01	.00	.06*		
BSQ	.62	.03	.53***		
Body Concern	.633	.05	.38***		
Thinness	.01	.04	.00		
Shortness	-.01	.03	-.01		
Facial Appearance	-.01	.04	-.01		

*Note*. * p < .05.

*** *p* < .001.

## Discussion

The current study represents the first validation of the English-translated version of the Negative Physical Self Scale (NPSS) in a sample of North American Asian women who were specifically living in Canada at the time of study recruitment. This research is significant as the English version of the NPSS has the potential to serve as a highly suitable and sensitive measure of body dissatisfaction (BD) specifically for this sub-population. When compared to previous research, the current findings did not support the four-factor structure proposed by Ly and colleagues [43] for a primarily non-Asian North American population. However, a second-order multidimensional model demonstrated good fit, aligning with the original NPSS validated within a Chinese sample [20]. This second-order model revealed that Body Concern, Thinness, Shortness, and Facial Appearance were second-order factors, each comprising three sub-dimensions encompassing cognition-affect, behavior, and projection. In the second-order multidimensional model, two items, namely Item 10 ("I try my best to be aware of my diet, in order to gain weight") and Item 19 ("I pay close attention to my weight"), were excluded due to factor loadings below the threshold of .60. Our findings indicate nuanced differences in the presentation of BD among Asian-identifying North American females compared to a predominantly Caucasian sample of North American women. Moreover, these results contrast with those reported by Wang et al. [47], who found equal factor structure and loadings of the NPSS across male ethnic groups. Thus, our findings suggest that the experience and perception of BD among Asian-identifying women living in Canada differ from Caucasian samples in terms of their multifaceted nature and complexity. These findings underscore the necessity for the utilization of assessment measures that are ethnically and culturally sensitive when working with minority populations.

As previously mentioned, Item 19 of the NPSS, which pertains to attention to weight, was excluded during the current analyses due to low factor loading. Notably, this specific item was also removed in Wang et al.’s [47] examination of the NPSS among North American males. Taken together, these results may indicate that, in North America, there is a greater emphasis on body shape as opposed to weight across genders. While previous studies have explored the intricate relationship between body shape, weight, and BD (e.g., [57]), further research is warranted to investigate whether body shape and weight play distinct roles in predicting BD across diverse cultures and ethnicities.

The Body Concern subscale demonstrated high convergence with other measures of BD suggesting that the subscale captures similar constructs and is a reliable assessment of BD. This finding suggests that the Body Concern subscale could be used in isolation as a measure of BD among Asian North American females. Moreover, the shorter length of the Body Concern subscale provides an advantage over other measures of BD traditionally used in clinical practice such as the BSQ. Previous studies (e.g., [65]) have called for shorter BD measures that can save time, and reduce respondent burden while still providing reliable and valid assessments of BD in clinical practice. Overall, the four subscales of the NPSS demonstrated high internal consistency, with Cronbach’s alpha values nearly identical to those reported by Ly et al. [43] and higher than those found by Chen et al. [20]. This supports the high reliability of the constructs being measured and suggests that the NPSS is consistently comparable to other Western measures of BD in an Asian sample.

The significant associations observed among all NPSS subscales indicate that they measure similar underlying constructs. Particularly, the Body Concern subscale showed the strongest association with the Facial Appearance subscale, suggesting that both body and facial dissatisfaction are important aspects of negative self-perception. This finding aligns with previous literature noting a greater emphasis on facial appearance in Asian cultures and greater concern with overall body shape in Western cultures [29]. Additionally, research conducted with Chinese, Malaysian, and Australian women has reported positive relationships between BD and dissatisfaction with weight, shape, and facial appearance [12]. These findings suggest that Asian women residing in North America may display similar levels of concern over both facial and body appearance, rather than a focus on single features.

The hierarchical regression analysis revealed that the NPSS accounted for a small but significant amount of variance in predicted scores on the EDE-Q. This supports the incremental validity of the NPSS, indicating that it provides unique information in understanding BD in a sample of North American Asian women. Notably, the Body Concern subscale was the only subscale that significantly contributed to the model, suggesting that the other NPSS subscales (Shortness, Thinness, and Facial Appearance) may not be as relevant to EDE-Q scores. These findings, combined with the strong convergent validity of the Body Concern subscale with other popular measures of BD, support the notion that the Body Concern subscale could be used as a standalone measure of BD in Asian women.

As the current study investigated BD in Asian women living in North America, it is important to consider the impact of immigration, acculturation, and sociocultural differences on body image. Interestingly, time living in North America was not associated with participants’ scores on any of the measures of BD, a finding which differs from the existing literature on acculturation [18, 66, 67]. Previous research has identified a positive relationship between acculturation, BD, and risk of developing an eating disorder in Chinese women [67]. Other investigations have found that, over the course of living in the United States for three months, female Japanese immigrants (on average) gained weight, perceived themselves as thinner, and overall showed decreased BD [68]. The researchers proposed that their findings could be attributed to greater exposure to larger bodies in the United States, making the Japanese participants perceive themselves as relatively thinner. In the present study, it is plausible that participants may have quickly assimilated Western body ideals and been exposed to Western media prior to arriving in North America. As a result, they may experience similar levels of BD compared to Asian individuals who have spent a longer duration in North America.

### Limitations and future research

The present study has several limitations that should be acknowledged. Firstly, the sample consisted of 18-25-year-old females, which may not fully represent the broader population. This age range is characterized by unique developmental and sociocultural factors that may influence body image and BD differently compared to other age groups. Another notable limitation is the homogeneity of our participant sample, comprised entirely of undergraduate students. The homogeneity of our sample may limit the extent to which our results can be extrapolated to a broader population. Additionally, the reliance on undergraduate students as participants may introduce a potential bias in the results based on shared participant characteristics. Future research endeavours should consider expanding the participant pool to include a more heterogeneous sample, encompassing individuals from various educational backgrounds and life stages. Furthermore, it is important to note that the present study did not directly measure acculturation and we did not disaggregate place of origin beyond what is presented in Table 1. Subsequent cross-cultural validation studies are encouraged to incorporate assessments of acculturation to enhance their investigations and our broader understanding of this issue as well as investigate differences based on specific places of origin. Regarding the interpretation of correlations between BMI and related measures, it should be noted that participants self-reported their height and weight measurements and may lack precision. Additionally, it should be noted that while the BSQ and EDE-Q were chosen as widely accepted measures to establish convergent validity, the psychometric properties of these measures have not been explicitly established in a validation study using Asian North American female samples. Furthermore, the BSQ and EDE-Q do not encompass all features examined within the NPSS (e.g., facial appearance and shortness). Future studies should aim to explicitly validate these measures among ethnic subpopulations including Asian North American females. A final consideration is the language used in discussing race, culture, and acculturation in this study. The measures employed (NPSS [20]; EDE-Q [26]; BSQ [2]) and much of the referenced literature were developed several years ago. The authors aimed to use the original descriptors of ethnicity reported by other researchers throughout the paper. As a result, some of the wording regarding body image and BD may be viewed as antiquated and fail to capture the cultural progression that has taken place over this time. With the increasing prevalence of multiracialism, acculturation, and assimilation, individuals may identify with multiple cultures or the culture of the country they reside in, rather than solely with their native culture. Future studies should consider updating the terminology used to address ethnicity and culture to reflect these advancements.

## Conclusion

In conclusion, this study represents the first examination of the English-translated version of the NPSS in a female Asian North American population. The findings did not support the four-factor model proposed by Ly et al. [20], which was based on a predominantly non-Asian North American population. However, the NPSS demonstrated high internal consistency and strong convergent validity between its subscales. The Body Concern subscale, in particular, shows promise as a measure of BD among Asian females in both clinical and research settings, given its strong convergence with other widely used measures of BD. Overall, the English-translated NPSS appears to be a valid and reliable measure of body dissatisfaction in the population of Asian North American women.
